# Effects of *Pholiota microspora* basal stipe powder on growth and development of *Tenebrio molitor*

**DOI:** 10.3389/finsc.2026.1916272

**Published:** 2026-09-10

**Authors:** Tong Mu, Ziyan Jiang, Honghong Hu, Yi Wang, Yikun Jia, ZhiHong Sun, Xiaolong He

**Affiliations:** 1 School of Life Sciences, Yan’an University/Yan’an Key Laboratory of Ruminant Molecular Cell Breeding, Yan’an, China; 2 School of Life Sciences, Yan’an Modern Sheep Industry Engineering Technology Research Center, Yan’an, China

**Keywords:** basal stipe powder, growth, insect meals, *Pholiota microspora*, *Tenebrio molitor*

## Abstract

**Introduction:**

*Pholiota microspora* basal stipe powder is a major by-product of mushroom harvesting and is rich in nutrients and bioactive compounds, making it a potential alternative feed ingredient for insect production. However, its effects on the growth and development of *Tenebrio molitor* remain unclear.

**Methods:**

five experimental diets were formulated by replacing wheat bran with *P.microspora* basal stipe powder at substitution levels of 0%, 25%,50%, 75%, and 100%. Growth and developmental parameters were assessed throughout the larval, pupal, and adult stages.

**Results:**

Larval body length, width, and weight increased with instar in all groups, and body length was strongly correlated with body width (r=0.93-0.94). The control group generally exhibited the greatest larval body size and weight, whereas the 100% substitution group showed the lowest values. Differences among treatments were minimal during the 6th-8th instars. From the 9th to 15th instars, larval growth in the 25% substitution group was comparable to that in the control group but significantly greater than that in the 50%, 75%, and 100% substitution groups (*P*<0.05). Larval duration increased significantly with substitution level (*P*<0.05), with the 100% substitution group requiring 34 more days to complete larval development than the control group. No significant differences were observed among groups in larval mortality, pupation rate, pupal duration, sex ratio, and eclosion rate (*P*>0.05). Pupal weight decreased as the substitution level increased. Although pupal weight in the 25% substitution group was significantly lower than that in the control group, it was significantly higher than that in the 50%, 75%, and 100% substitution groups (*P*<0. 05). Adult body weight was significantly lower in all substitution groups than in the control group (*P*<0.05).

**Discussion:**

Replacing up to 25% of wheat bran with *P. microspora* basal stipe powder had minimal effects on T.molitor growth and development, whereas higher levels impaired larval growth and reduced pupal and adult weights. Therefore, a 25% substitution level appears to be the most suitable among the tested levels for incorporating *P.microspora* basal stipe powder into *T.molitor* diets.

## 1 Introduction

Insects are widely recognized as high-quality alternative protein sources for food and feed and as important contributors to sustainable food systems (1, 2). *Tenebrio molitor* (Coleoptera: Tenebrionidae) is a common stored-product pest and one of the most widely reared insect species worldwide (3). Under Regulation (EU) 2015/2283 of the European Parliament and of the Council, several insect-derived products, including products derived from *T. molitor*, have been authorized as novel foods for human consumption in the European Union (4). *T. molitor* has a high crude protein content, ranging from 40.2% to 63.3% on a dry-matter basis, and a well-balanced amino acid profile that includes essential amino acids such as lysine, phenylalanine, threonine, and valine (5, 6). It is also a valuable source of unsaturated fatty acids, vitamins, and minerals (7). These nutritional characteristics support the use of *T. molitor* as a protein-rich feed ingredient and a potential source of functional food and health-product ingredients (8–10). In addition, its polyphagous feeding behavior, environmental adaptability, high reproductive capacity, and ease of rearing make it suitable for the bioconversion of organic waste, the mass rearing of natural enemies, and pesticide residue detection (11, 12).

*T. molitor* larvae are generally reared on cereal-based diets, in which starch serves as an important energy source for growth and development (13). When starch is limited, larvae can adjust their feed intake and derive energy form other nutrients, including dietary protein and digestible fiber fractions (14). Their nutritional plasticity and polyphagous feeding behavior enable them to utilize a wide range of agricultural and food-processing by-products. Studies of starch-rich substrates have shown that wheat bran supports favorable larval growth and is therefore one of the most commonly used basal feeds (14, 15). Various agricultural and food-processing by-products have been evaluated as partial or complete substitutes for conventional feeds. Oonincx et al. (16) reported that diets formulated with selected by-products supported larval survival and development. Kim et al. (17) evaluated orange peel, cabbage, king oyster mushroom, and shiitake mushroom substrates as wheat bran replacements and found that shiitake mushroom substrate supported larval rearing at replacement levels of 40% and 50%. However, the capacity of *T. molitor* to utilize diverse substrates does not mean that all substrates are nutritionally adequate or safe. For example, Janković-Tomanić et al. (18) reported that larvae fed zearalenone-contaminated grain showed no significant difference in survival but exhibited reduced body weight and locomotor activity. Therefore, although the dietary flexibility of *T. molitor* offers considerable potential for the bioconversion of agricultural by-products and the development of low-cost rearing systems, the nutritional suitability and physiological effects of each alternative substrate must be evaluated.

The high cost of conventional feed remains a major constraint on the large-scale production of *T. molitor*. Mushroom production residues may serve as low-cost feed resources because they are generated in large quantities and are often available year-round. In particular, spent mushroom substrates have been reported to retain approximately 75-85% of their original nutrients after mushroom harvesting (19). Previous studies have shown that mushroom-derived substrates can be incorporated into *T. molitor* diets, although their effects on larval performance vary according to mushroom species and dietary inclusion level (17, 20). Therefore, the suitability and optimal inclusion level of each mushroom-derived ingredient should be evaluated separately.

In addition to spent cultivation substrates, mushroom harvesting and processing generate other potentially valuable by-products. The basal 1–3 cm of the *Pholiota microspora* stipe, unlike the more tender upper portion of the fruiting body, is unsuitable for commercial sale and is typically discarded during production. *P. microspora* fruiting bodies contains carbohydrates, proteins, dietary fiber, polysaccharides, minerals, vitamins, and amino acids (21–23), some of which may also be retained in the discarded basal stipe portions. The potential retention of these nutrients warrants evaluation of this by-product as an alternative feed ingredient for *T. molitor*. However, its suitability for larval rearing and the appropriate dietary inclusion level have not yet been investigated. This study evaluated diets in which wheat bran was partially replaced with different proportions of dried *P. microspora* basal stipe powder and assessed their effects on larval growth, development, survival, and sex ratio. The objective aimed to evaluate the feasibility of using this by-product as an alternative feed ingredient and to determine its optimal inclusion level, thereby providing a basis for reducing rearing costs and valorizing mushroom-production residues.

## 2 Materials and methods

### 2.1 Experimental insects and rearing conditions

Tenth-instar *T. molitor* larvae were purchased from a commercial insect supplier in China and maintained under laboratory conditions until pupation and adult emergence. The emerged adults were allowed to mate and oviposit, and the resulting eggs were collected and incubated to establish the first laboratory generation (F1). F1 larvae were reared to adulthood, and the resulting adults were allowed to mate and oviposit to produce the second laboratory generation (F2). Uniformly sized fifth-instar F2 larvae were selected for the experiment. All developmental stages were maintained in a BluePard programmable climate chamber at 25 °C and 60% relative humidity. Dried basal stipe powder of *P. microspora* was supplied by Yan’an Junjian Co., Ltd. Wheat bran, corn flour, soybean meal, vitamin premix, and salt were obtained from a commercial feed supplier. All feed ingredients were confirmed to be free of pesticide residues.

### 2.2 Experimental design

**Figure 1:**
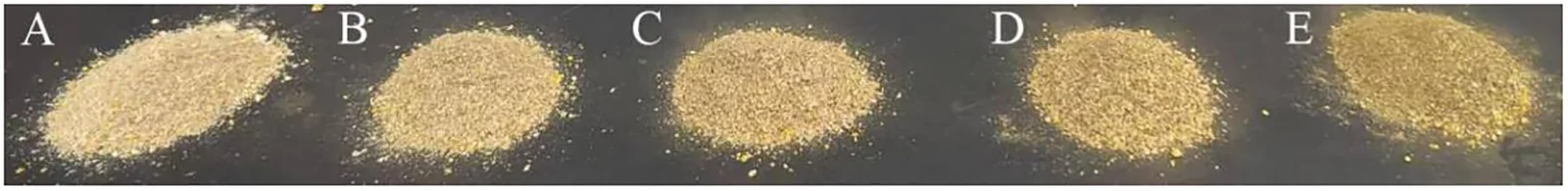
Experimental diet samples with different replacement levels of wheat bran by *P. microspora* basal stipe powder. Groups **(A–E)** represent substitution levels of 0%, 25%, 50%, 75%, and 100% of *P. microspora* basal stipe powder, respectively.

**Figure 2:**
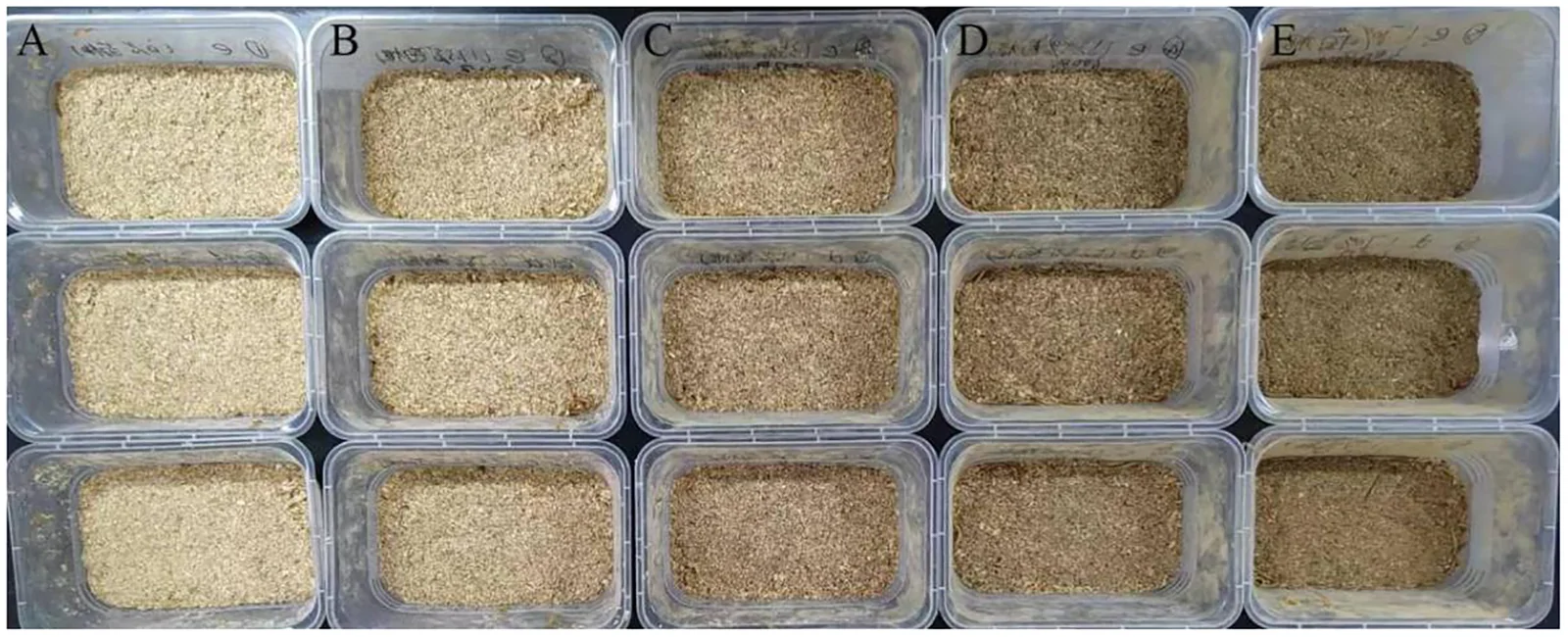
Rearing conditions of *T. molitor* larvae. Groups **(A–E)** represent substitution levels of 0%, 25%, 50%, 75%, and 100% of *P. microspora* basal stipe powder, respectively.

To investigate the effects of replacing wheat bran with varying proportions of *P. microspora* basal stipe powder on the growth and development of *T. molitor*, five dietary treatments were established, each with three biological replicates per treatment. Each replicate contained 150 healthy, uniformly sized fifth-instar larvae. The composition and appearance of the experimental diets are presented in **Table 1** and **Figure 1**, respectively, while the larval feeding conditions are illustrated in **Figure 2**.

**Table 1:** Composition of the experimental diets for *T. molitor*.

Ingredient	Content (%)				
	Control	A	B	C	D
wheat bran	70	52.5	35	17.5	—
*P. microspora* basal stipe powder	—	17.5	35	52.5	70
corn flour	24	24	24	24	24
soybean meal	5	5	5	5	5
compound vitamin	0.5	0.5	0.5	0.5	0.5
salt	0.5	0.5	0.5	0.5	0.5
total	100	100	100	100	100

Control, groups A, B, C, and D represent substitution levels of 0%, 25%, 50%, 75%, and 100% of P. microspora basal stipe powder, respectively,

### 2.3 The measurement indicators and methods

#### 2.3.1 Measurement of larval body length, width, and weight in *T. molitor*

Uniformly sized fifth-instar F2 *T. molitor* larvae were selected at the beginning of the experiment. Based on previous reports indicating that each larval instar of *T. molitor* lasts approximately 10 days under comparable rearing conditions (24), larval instars in the present study were determined at 10-day intervals. Accordingly, larval growth and developmental indices were measured at the 6th, 8th, 10th, 12th, 14th, and 15th instars. At each sampling point, body length and width were measured in 15 larvae randomly selected from each of the three biological replicates within each treatment. Body length was measured using a ruler, and body width was measured at the seventh abdominal segment. In addition, 30 larvae were randomly selected from each replicate and individually weighed using an MP200 electronic balance. The mean individual body weight was then calculated for each replicate.

#### 2.3.2 Determination of pupal weight, sex ratio and adult weight

Newly formed pupae were collected daily from each treatment and sexed based on the morphology of their genital papillae under a stereomicroscope (25). Female and male pupae were then placed in separate plastic containers and maintained until adult emergence. The pupae and adults were weighed individually using an MP200A electronic balance with a readability of 0.001 g. The sex ratio was calculated for each treatment.

#### 2.3.3 Determination of larval duration and pupal duration

For each biological replicate, the time from larval hatching to 50% pupation and that from the first observed pupation to 50% adult emergence were recorded as larval and pupal durations, respectively (26). The durations were then averaged across three biological replicates.

#### 2.3.4 Determination of larval mortality, pupation rate and eclosion rate

For each treatment, the numbers of dead larvae, pupae and adult insects were counted to calculate larval mortality, pupation rate and eclosion rate as follows: larval mortality (%) = (dead larvae/total larvae) × 100; pupation rate (%) = (pupae/total larvae) × 100; eclosion rate (%) = (emerged adults/pupae) × 100.

#### 2.3.5 Data processing

Data were analyzed using R (version 4.3.3). Data manipulation and summary statistics were performed using the dplyr package. Normality and homogeneity of variances were assessed using the Shapiro-Wilk and Bartlett’s tests, respectively. Differences among groups were evaluated by one-way analysis of variance (ANOVA), followed by Tukey’s honestly significant difference (HSD) test for multiple comparisons. Statistical significance was set at *P* < 0.05. Line plots of body length, body width, and body weight across instars were generated separately for each treatment group using the ggplot2 package, and significant differences were annotated using the ggsignif package. Linear relationships between body length and body width were assessed separately within each treatment group.

## 3 Results

### 3.1 Effects of *P. microspora* basal stipe powder supplementation on larval growth

**Figure 3:**
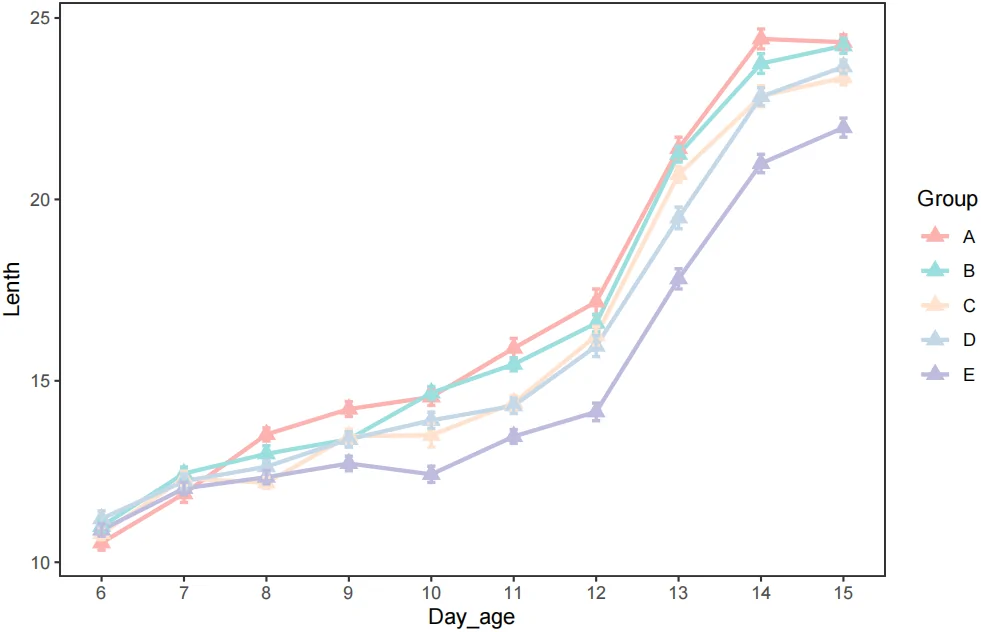
Body length of *T. molitor* larvae at different instars under different dietary treatments. Groups A–E represent wheat bran replacement levels of 0%, 25%, 50%, 75%, and 100% of *P. microspora* basal stipe powder, respectively.

**Figure 4:**
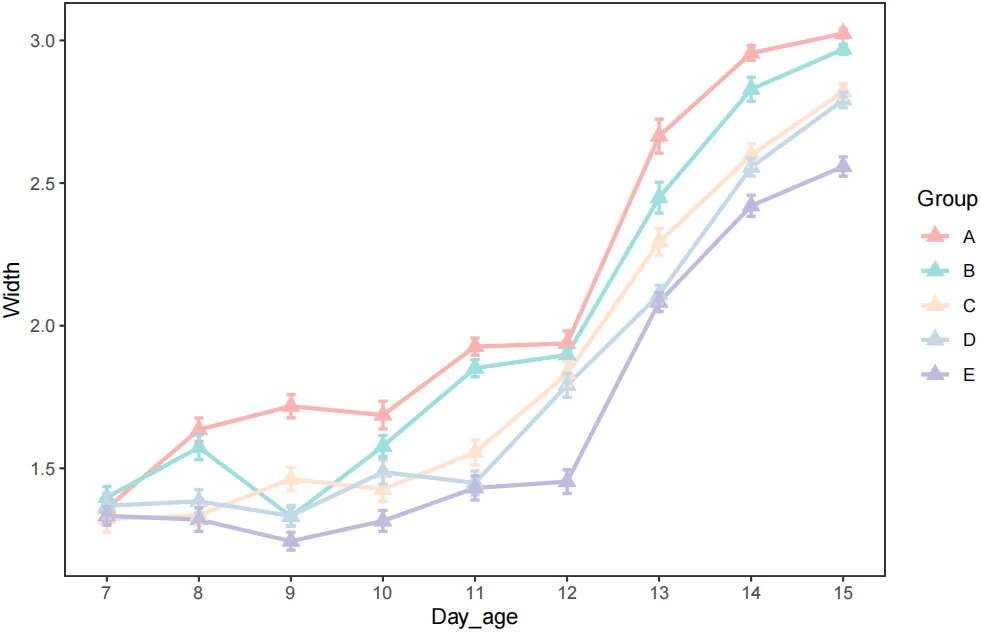
Body width of *T.molitor* larvae at different instars under different dietary treatments. Groups A–E represent wheat bran replacement levels of 0%, 25%, 50%, 75%, and 100% of *P. microspora* basal stipe powder, respectively.

**Figure 5:**
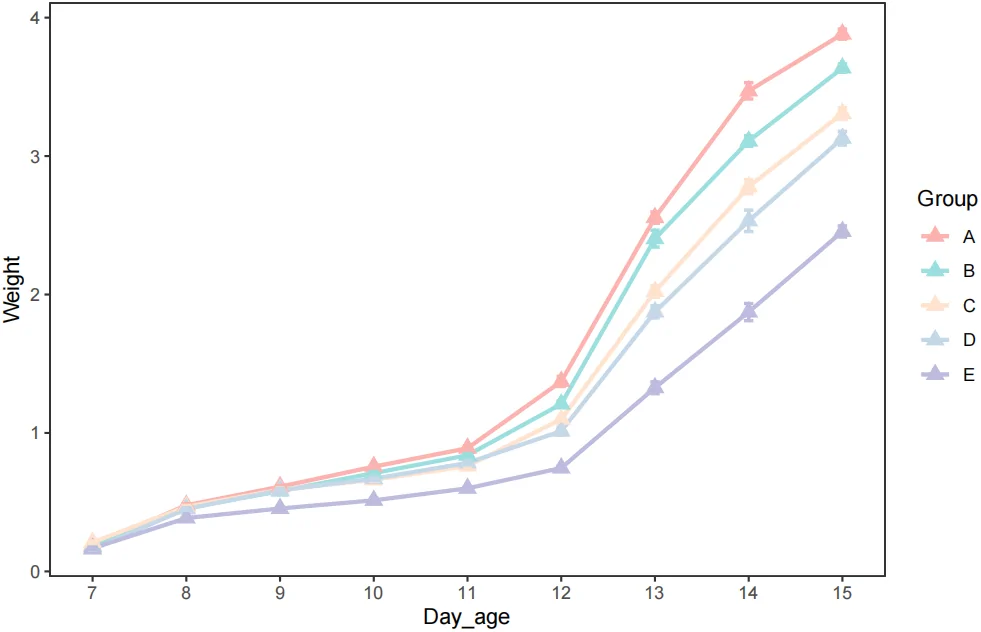
Body weight of *T. molitor* larvae at different instars under different dietary treatments. Groups A–E represent wheat bran replacement levels of 0%, 25%, 50%, 75%, and 100% of *P. microspora* basal stipe powder, respectively.

**Figure 6:**
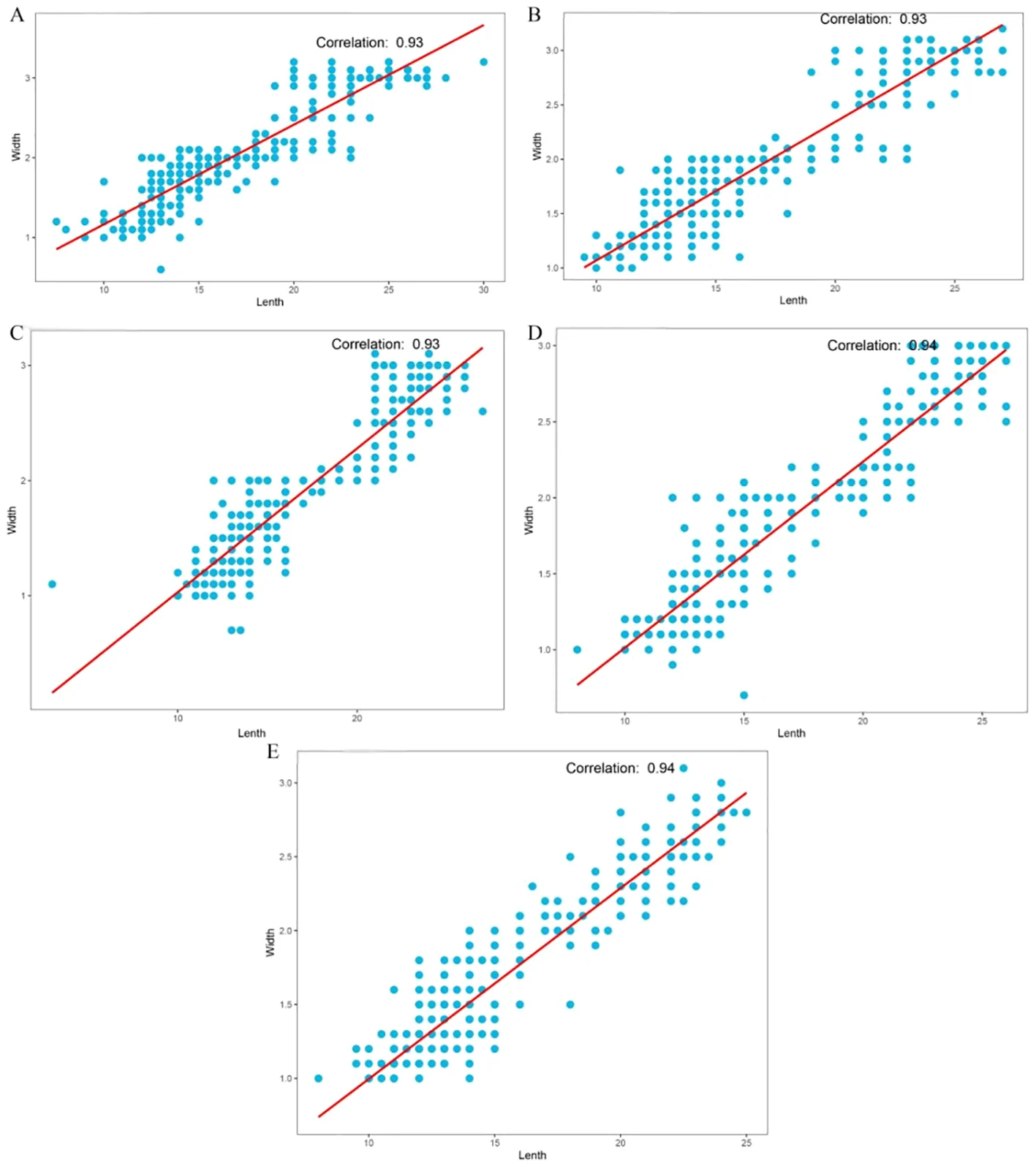
Correlation between body length and width of *T. molitor* larvae fed diets containing different levels of *P. microspora* basal stipe powder. Groups **(A–E)** represent wheat bran replacement levels of 0%, 25%, 50%, 75%, and 100% with *P. microspora* basal stipe powder, respectively.

Across all dietary treatments, larval body length, width, and weight increased with advancing instar (**Table 2**, **Figures 3**–**5**). The coefficients of variation for these growth traits were presented in **Supplementary Table 1**. Body length was strongly and positively correlated with body width within each treatment group (r = 0.93-0.94; **Figure 6**). Body length ranged from 10.50-24.42 cm in the control group and from 11.00-24.23, 10.80-23.36, 11.20-23.66, and 10.90-21.98 cm in Groups A-D, respectively. The corresponding ranges for body width were 1.36-3.02, 1.33-2.97, 1.32-2.82, 1.33-2.79, and 1.32-2.56 cm, while those for body weight were 0.19-3.88, 0.18-3.64, 0.21-3.31, 0.16-3.13, and 0.17-2.46 g, respectively. Differences among treatments were small during the 6th-8th instars. During the 9th-11th instars, all three traits in the 25% replacement group were comparable to those in the control and were generally higher than those in the 50%, 75%, and 100% replacement groups. During the 12th-15th instars, the 25% replacement group remained comparable to the control, whereas all three traits in the 100% replacement group were significantly lower than those in the control (P < 0.05). These results indicate that replacing 25% of wheat bran with P. microspora basal stipe powder did not adversely affect larval growth.

**Table 2:** Effects of replacing wheat bran with *P. microspora* basal stipe powder on the growth of *T. molitor* larvae.

Instars (day)	Trait	Control	Group A	Group B	Group C	Group D
6	Length/cm	10.50 ± 1.34	11.00 ± 1.06	10.80 ± 1.13	11.20 ± 1.44	10.90 ± 1.14
	Width/cm	——	——	——	——	——
	Weight/g	——	——	——	——	——
7	Length/cm	11.90 ± 1.62	12.40 ± 1.21	12.3 ± 1.32	12.2 ± 1.43	12.00 ± 1.16
	Width/cm	1.36 ± 0.34	1.40 ± 0.26	1.32 ± 0.32	1.37 ± 0.30	1.33 ± 0.22
	Weight/g	0.19 ± 0.02^ab^	0.18 ± 0.03^ab^	0.21 ± 0.03^a^	0.16 ± 0.02^b^	0.17 ± 0.02^b^
8	Length/cm	13.52 ± 1.22^a^	12.99 ± 1.48^ab^	12.18 ± 1.02^c^	12.63 ± 1.13^bc^	12.34 ± 1.23^bc^
	Width/cm	1.64 ± 0.28^a^	1.57 ± 0.29^a^	1.34 ± 0.24^b^	1.38 ± 0.28^b^	1.32 ± 0.28^b^
	Weight/g	0.48 ± 0.04^a^	0.46 ± 0.01^a^	0.47 ± 0.03^a^	0.45 ± 0.03^a^	0.38 ± 0.01^b^
9	Length/cm	14.22 ± 1.38^a^	13.38 ± 1.41^b^	13.47 ± 1.32^ab^	13.39 ± 1.43^b^	12.72 ± 1.35^b^
	Width/cm	1.72 ± 0.27^a^	1.33 ± 0.22^bc^	1.46 ± 0.28^b^	1.33 ± 0.24^bc^	1.24 ± 0.21^c^
	Weight/g	0.61 ± 0.03^a^	0.58 ± 0.03^a^	0.59 ± 0.04^a^	0.59 ± 0.04^a^	0.45 ± 0.03^b^
10	Length/cm	14.55 ± 1.56^a^	14.67 ± 1.19^a^	13.50 ± 2.20^b^	13.91 ± 1.50^ab^	12.42 ± 1.50^c^
	Width/cm	1.69 ± 0.33^a^	1.58 ± 0.25^a^	1.43 ± 0.29^a^	1.49 ± 0.28^a^	1.60 ± 1.91^b^
	Weight/g	0.76 ± 0.04^a^	0.71 ± 0.04^ab^	0.66 ± 0.04^b^	0.67 ± 0.04^b^	0.51 ± 0.04^c^
11	Length/cm	15.90 ± 1.80^a^	15.46 ± 1.22^a^	14.39 ± 1.37^b^	14.31 ± 1.42^b^	13.47 ± 1.29^c^
	Width/cm	1.93 ± 0.20^a^	1.85 ± 0.20^a^	1.56 ± 0.29^b^	1.45 ± 0.28^b^	1.43 ± 0.29^b^
	Weight/g	0.89 ± 0.05^a^	0.84 ± 0.04^ab^	0.76 ± 0.05^c^	0.78 ± 0.04^bc^	0.60 ± 0.03^d^
12	Length/cm	17.18 ± 2.38^a^	16.59 ± 1.62^ab^	16.24 ± 1.73^ab^	15.96 ± 1.93^b^	14.14 ± 1.63^c^
	Width/cm	1.94 ± 0.29^a^	1.90 ± 0.14^a^	1.83 ± 0.28^a^	1.79 ± 0.28^a^	1.45 ± 0.28^b^
	Weight/g	1.37 ± 0.11^a^	1.21 ± 0.07^b^	1.10 ± 0.04^c^	1.01 ± 0.09^c^	0.75 ± 0.05^d^
13	Length/cm	21.40 ± 2.11^a^	21.24 ± 1.43^a^	20.69 ± 1.38^a^	19.49 ± 2.00^b^	17.81 ± 1.87^c^
	Width/cm	2.66 ± 0.40^a^	2.45 ± 0.36^b^	2.29 ± 0.32^b^	2.11 ± 0.22^c^	2.08 ± 0.22^c^
	Weight/g	2.56 ± 0.12^a^	2.40 ± 0.18^a^	2.02 ± 0.13^b^	1.87 ± 0.13^b^	1.33 ± 0.13^c^
14	Length/cm	24.42 ± 1.84^a^	23.74 ± 1.82^ab^	22.84 ± 1.93^b^	22.83 ± 1.65^b^	20.99 ± 1.70^c^
	Width/cm	2.96 ± 0.18^a^	2.83 ± 0.28^a^	2.60 ± 0.27^b^	2.56 ± 0.21^bc^	2.42 ± 0.25^c^
	Weight/g	3.47 ± 0.18^a^	3.11 ± 0.11^b^	2.78 ± 0.15^c^	2.53 ± 0.23^d^	1.87 ± 0.19^e^
15	Length/cm	24.33 ± 1.37^a^	24.23 ± 1.38^a^	23.36 ± 1.33^b^	23.66 ± 1.25^ab^	21.98 ± 1.76^c^
	Width/cm	3.02 ± 0.11^a^	2.97 ± 0.12^a^	2.82 ± 0.18^b^	2.79 ± 0.18^b^	2.56 ± 0.23^c^
	Weight/g	3.88 ± 0.11^a^	3.64 ± 0.09^b^	3.31 ± 0.13^c^	3.13 ± 0.15^d^	2.46 ± 0.12^e^

In the control and groups A, B, C, and D, *P. microspora* basal stipe powder replaced 0%, 25%, 50%, 75%, and 100% of the wheat bran, respectively. Within each row, values followed by different lowercase letters differ significantly (*P* < 0.05).

### 3.2 Effects of *P. microspora* basal stipe powder supplementation on larval mortality and duration

Replacement of wheat bran with *P. microspora* basal stipe powder did not significantly affect larval mortality (**Table 3**), although mortality was numerically higher in the replacement groups (9.11-10.45%) than in the control (7.67%). In contrast, Larval duration increased significantly with increasing replacement level (*P* < 0.05), from 144.67 days in the control to 178.67 days at 100% replacement, representing a 34-day prolongation.

**Table 3:** Effects of replacing wheat bran with different levels of *P. microspora* basal stipe powder on the larval mortality and duration.

Treatment	Larval mortality (%)	Larval duration (day)
Control	7.67 ± 1.00	144.67 ± 0.58^d^
Group A	9.78 ± 2.14	148.33 ± 0.58^cd^
Group B	10.00 ± 3.71	152.67 ± 4.16^c^
Group C	9.11 ± 1.68	160.00 ± 2.65^b^
Group D	10.45 ± 4.02	178.67 ± 3.21^a^

In the control and groups A, B, C, and D, *P. microspora* basal stipe powder replaced 0%, 25%, 50%, 75%, and 100% of the wheat bran, respectively. Within each row, values followed by different lowercase letters differ significantly (*P* < 0.05).

### 3.3 Effects of *P. microspora* basal stipe powder supplementation on pupation rate, pupal duration and sex ratio of *T. molitor*

Replacing wheat bran with *P. microspora* basal stipe powder did not significantly affect pupation rate, pupal duration, and sex ratio of *T. molitor* (*P* > 0.05). Nevertheless, pupation rate and pupal duration showed a decreasing trend as the replacement level increased, whereas the sex ratio remained relatively stable among treatments (**Table 4**).

**Table 4:** Effects of replacing wheat bran with different levels of *P. microspora* basal stipe powder on the pupation rate, pupal duration and sex ratio.

Treatment	Pupation rate (%)	Pupal duration (day)	Sex ratio
Control	92.33 ± 1.00	10.00 ± 0.00	0.94 ± 0.16
Group A	90.22 ± 2.14	9.00 ± 0.00	1.08 ± 0.15
Group B	90.00 ± 3.71	9.67 ± 2.89	0.94 ± 0.15
Group C	90.89 ± 1.68	8.67 ± 0.58	1.06 ± 0.44
Group D	89.55 ± 4.02	8.00 ± 2.65	0.93 ± 0.20

Control, groups A, B, C, and D represent substitution levels of 0%, 25%, 50%, 75%, and 100% of P. microspora basal stipe powder, respectively.

### 3.4 Effects of *P. microspora* basal stipe powder supplementation on pupal weight, adult weight, and eclosion rate

**Figure 7:**
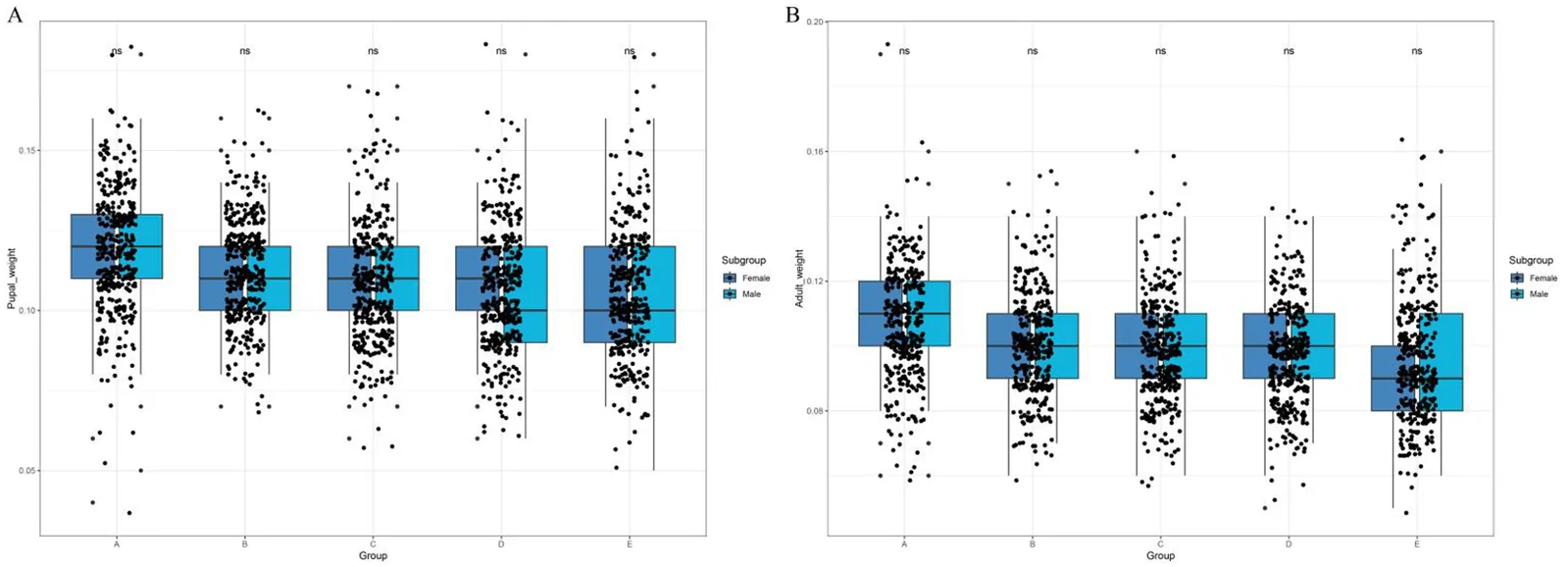
Sex-specific differences in the pupal and adult weights of *T. molitor* under different dietary treatments. Groups A–E represent wheat bran replacement levels of 0%, 25%, 50%, 75%, and 100% with *P. microspora* basal stipe powder, respectively. **(A)** Sex-specific differences in the pupal weights of T. molitor under different dietary treatments. **(B)** Sex-specific differences in the adult weights of T. molitor under different dietary treatments.

Replacing wheat bran with *P. microspora* basal stipe powder significantly reduced the pupal and adult weights of *T. molitor* but did not affect eclosion rate (**Tables 5** and **6**). Pupal weight decreased significantly with increasing replacement level, from 0.118 g in the control to 0.105 g at 100% replacement (*P* < 0.05). Adult weight similarly declined from 0.106 g in the control to 0.095 g at 100% replacement *(P* < 0.05). Eclosion rate decreased from 10.00% to 8.00%, but this difference was not significant (*P* > 0.05). Female and male pupal and adult weights showed similar declining patterns across replacement levels, with no significant sex-specific differences detected (**Figure 7**).

**Table 5:** Effects of replacing wheat bran with different levels of *P. microspora* basal stipe powder on pupal weight.

Treatment	Pupal weight (g)	Female pupal weight (g)	Male pupal weight (g)
Control	0.118 ± 0.02^a^	0.119 ± 0.02^a^	0.118 ± 0.02^a^
Group A	0.113 ± 0.02^b^	0.114 ± 0.02^b^	0.112 ± 0.02^b^
Group B	0.108 ± 0.02^c^	0.106 ± 0.02^c^	0.110 ± 0.02^bc^
Group C	0.106 ± 0.02^c^	0.107 ± 0.02^c^	0.106 ± 0.02^c^
Group D	0.105 ± 0.02^c^	0.104 ± 0.02^c^	0.106 ± 0.02^c^

In the control and groups A, B, C, and D, *P. microspora* basal stipe powder replaced 0%, 25%, 50%, 75%, and 100% of the wheat bran, respectively. Within each row, values followed by different lowercase letters differ significantly (*P* < 0.05).

**Table 6:** Effects of replacing wheat bran with different levels of *P. microspora* basal stipe powder on adult weight, and eclosion rate.

Treatment	Eclosion rate (%)	Adult weight (g)	Female adult weight (g)	Male adult weight (g)
Control	10.00 ± 0.00	0.106 ± 0.02^a^	0.106 ± 0.02^a^	0.107 ± 0.02^a^
Group A	9.00 ± 0.00	0.100 ± 0.02^b^	0.101 ± 0.02^b^	0.100 ± 0.01^b^
Group B	9.67 ± 2.89	0.098 ± 0.02^bc^	0.097 ± 0.02^bc^	0.098 ± 0.02^b^
Group C	8.67 ± 0.58	0.098 ± 0.02^bc^	0.097 ± 0.01^bc^	0.098 ± 0.02^b^
Group D	8.00 ± 2.65	0.095 ± 0.02^c^	0.094 ± 0.02^c^	0.096 ± 0.02^b^

In the control and groups A, B, C, and D, *P. microspora* basal stipe powder replaced 0%, 25%, 50%, 75%, and 100% of the wheat bran, respectively. Within each row, values followed by different lowercase letters differ significantly (*P* < 0.05).

## 4 Discussion

The valorization of edible mushroom cultivation by-products as insect feed may reduce resource waste while diversifying feed sources for *T. molitor*. In this study, we evaluated the effects of dietary *P. microspora* basal stipe powder on the growth of *T. molitor* to assess its potential as an insect-feed ingredient. Larval body length, body width, and body weight increased with age in all treatment groups. However, the larval body length and body weight recorded in the present study differed from those reported by Riaz et al. (26) and Hardouin et al. (27). These differences may be attributable to variations in experimental conditions, including diet formulation, larval developmental stage, rearing density, temperature, and measurement procedures.

Larval growth parameters in the 25% replacement group did not differ significantly from those in the control group and were generally higher than those in the 50%, 75%, and 100% replacement groups. The reduced growth observed at higher replacement levels may reflect the increasing proportion of *P. microspora* basal stipe powder in the diet, which could have affected nutrient utilization and feed intake. Because *P. microspora* basal stipe powder had a smaller particle size than wheat bran, increasing its proportion in the diet may have altered dietary physical properties. Previous studies have suggested that reduced feed particle size can influence bulk density and nutrient digestibility (28). Higher replacement levels may also have altered the texture, color, and odor of the diet, potentially reducing feed acceptability and larval feed intake (29). In addition, the fiber content of *P. microspora* basal stipe powder may have contributed to reduced growth at high replacement levels, as elevated dietary fiber levels can reduce digestibility and impair larval development (30–33). Therefore, the lower growth performance observed at high replacement levels may be associated with reduced nutrient availability and feed utilization. However, because feed intake, diet physical properties, and nutrient digestibility were not measured, these proposed mechanisms remain speculative and require further investigation.

Replacement of wheat bran with *P. microspora* basal stipe powder did not significantly affect larval mortality, although mortality was numerically higher in the replacement groups (9.11-10.45%) than in the control group (approximately 7%). Mortality across all groups was higher than the approximately 5% previously reported by Montalbán et al. (34). Li et al. (20) reported that complete replacement of conventional feed with *Lentinula edodes* spent mushroom substrate significantly reduced larval survival. Similarly, Kim et al. (17) found that *Pleurotus eryngii* spent mushroom substrate could replace conventional feed without affecting larval survival, although larval growth was reduced. Riaz et al. (26) also reported the lowest mortality in *T. molitor* larvae fed wheat bran, which is consistent with our study. Overall, these findings suggest that the effects of mushroom-derived by-products on larval mortality depend on diet composition and rearing conditions. Low-to-moderate replacement of wheat bran appears feasible, whereas high replacement levels may impair larval growth and survival. Possible explanations include the fiber content and other poorly digestible components of *P. microspora* basal stipe powder, which may reduce nutrient utilization (35). Further studies on digestive enzyme activity and gut microbiota are needed to clarify these mechanisms.

Larval duration increased significantly with increasing replacement levels of *P. microspora* basal stipe powder, consistent with the findings of Soomro et al. (36). This prolonged larval period may be attributable to nutritional limitations at higher basal stipe powder inclusion levels, which could delay larval development and pupation. Mushroom-derived residues may also contain phenolic compounds that inhibit fiber-degrading microorganisms and reduce fiber digestibility (15), thereby further limiting nutrient availability. However, this mechanism requires verification through analyses of phenolic compounds, nutrient digestibility, digestive enzyme activity, and gut microbial communities. Although replacement level did not significantly affect pupation rate, pupal duration, or eclosion rate, these parameters were generally numerically lower at higher replacement levels. No significant differences in pupal or adult weight were detected between sexes within any dietary treatment. These results indicate that low replacement levels may be biologically feasible, whereas high replacement levels may prolong the production cycle and reduce production efficiency.

From a practical perspective, replacing 25% of wheat bran with *P. microspora* basal stipe powder could reduce reliance on conventional feed ingredients and provide a productive use for mushroom cultivation residues. However, savings in wheat bran costs may be offset by residue processing and handling costs. In addition, higher replacement levels may prolong the production cycle and reduce production efficiency. The actual economic benefit of this substitution cannot yet be determined because feed intake, feed conversion efficiency, processing costs, and economic returns were not assessed in this study. Nevertheless, the results suggest that a 25% replacement level is a promising starting point, as it maintained larval growth performance. Further studies should evaluate processing methods, nutrient composition, and cost-benefit performance under commercial production conditions.

## 5 Conclusions

This study systematically evaluated the effects of replacing wheat bran with *P. microspora* basal stipe powder on the growth and development of *T. molitor*. The results indicate that *P. microspora* basal stipe powder can partially replace wheat bran in *T. molitor* diets. Larval body length, width, and weight at a 25% substitution ratio were comparable to those of the control group and significantly greater than those observed at substitution ratios of 50% or higher, indicating that 25% was a suitable substitution ratio among those tested. Higher substitution ratios significantly prolonged larval development and reduced pupal and adult weights. However, no significant differences were observed among treatment groups in larval mortality, pupation rate, pupal duration, sex ratio, and eclosion rate, indicating that *P. microspora* basal stipe powder had no adverse effects on the measured biological parameters. Overall, these findings support the feasibility of partially replacing wheat bran with *P. microspora* basal stipe powder in *T. molitor* diets, provided that the substitution ratio is appropriately controlled. Furthermore, incorporating this material into *T. molitor* diets may reduce production costs, improve economic returns, and promote the valorization of agricultural waste and the development of a circular economy.

## Data Availability

The original contributions presented in the study are included in the article/**Supplementary Material**, further inquiries can be directed to the corresponding author/s.
