# Supplementary material for: Effects of Pholiota microspora basal stipe powder on growth and development of Tenebrio molitor
Source: Front Insect Sci. 2026 Sep 10;6:1916272. doi: 10.3389/finsc.2026.1916272 (PMC13601258; doi:10.3389/finsc.2026.1916272)
Supplement: Supplementary file 1 [file Table1.docx]

Supplementary Material

# 1 Supplementary Tables

**Supplementary Table 1**. Coefficients of variation for the growth parameters of *T. molitor* larvae under different dietary treatments.

| **Instars (day)** | **Trait** | **Control** | **Group A** | **Group B** | **Group C** | **Group D** |
| --- | --- | --- | --- | --- | --- | --- |
| 6 | length | 12.76 | 9.64 | 10.46 | 12.86 | 10.46 |
|  | width | —— | —— | —— | —— | —— |
|  | weight | —— | —— | —— | —— | —— |
| 7 | length | 13.61 | 9.76 | 10.73 | 11.72 | 9.67 |
|  | width | 25.00 | 18.57 | 24.24 | 21.90 | 16.54 |
|  | weight | 10.53 | 16.67 | 14.29 | 12.50 | 11.76 |
| 8 | length | 9.02 | 11.39 | 8.37 | 8.95 | 9.97 |
|  | width | 17.07 | 18.47 | 17.91 | 20.29 | 21.21 |
|  | weight | 8.33 | 2.17 | 6.38 | 6.67 | 2.63 |
| 9 | length | 9.70 | 10.54 | 9.80 | 10.68 | 10.61 |
|  | width | 15.70 | 16.54 | 19.18 | 18.05 | 16.94 |
|  | weight | 4.92 | 5.17 | 6.78 | 6.78 | 6.67 |
| 10 | length | 10.72 | 8.11 | 16.30 | 10.78 | 12.08 |
|  | width | 19.53 | 15.82 | 20.28 | 18.79 | 119.38 |
|  | weight | 5.26 | 5.63 | 6.06 | 5.97 | 7.84 |
| 11 | length | 11.32 | 7.89 | 9.52 | 9.92 | 9.58 |
|  | width | 10.36 | 10.81 | 18.59 | 19.31 | 20.28 |
|  | weight | 5.62 | 4.76 | 6.58 | 5.13 | 5.00 |
| 12 | length | 13.85 | 9.76 | 10.65 | 12.09 | 11.53 |
|  | width | 14.95 | 7.37 | 15.30 | 15.64 | 19.31 |
|  | weight | 8.03 | 5.79 | 3.64 | 8.91 | 6.67 |
| 13 | length | 9.86 | 6.73 | 6.67 | 10.26 | 10.50 |
|  | width | 15.04 | 14.69 | 13.97 | 10.43 | 10.58 |
|  | weight | 4.69 | 7.50 | 6.44 | 6.95 | 9.77 |
| 14 | length | 7.53 | 7.67 | 8.45 | 7.23 | 8.10 |
|  | width | 6.08 | 9.89 | 10.38 | 8.20 | 10.33 |
|  | weight | 5.19 | 3.54 | 5.40 | 9.09 | 10.16 |
| 15 | length | 5.63 | 5.70 | 5.69 | 5.28 | 8.01 |
|  | width | 3.64 | 4.04 | 6.38 | 6.45 | 8.98 |
|  | weight | 2.84 | 2.47 | 3.93 | 4.79 | 4.88 |

Note: In the control and groups A, B, C, and D, *P. microspora* basal stipe powder replaced 0%, 25%, 50%, 75%, and 100% of the wheat bran, respectively.
